# Heterologous Production of Barnesin A, an NRPS–PKS Hybrid Containing a Rare Vinylogous Arginine Moiety

**DOI:** 10.1002/cbic.70305

**Published:** 2026-04-21

**Authors:** Sven Balluff, Marie Dayras, Christine Beemelmanns

**Affiliations:** ^1^ Antiinfectives from Microbiota Helmholtz Institute for Pharmaceutical Research Saarland Saarbrücken Germany; ^2^ PharmaScienceHub Faculty of Medicine Saarland University Saarbrücken Germany

**Keywords:** biosynthesis, heterologous expression, natural products, nonribosomal peptide, protease inhibitor, vinylogous amino acid

## Abstract

Natural products containing vinylogous amino acids are rarely found in nature and often possess significant biological activity. Barnesin A was the first NP reported from an anaerobic bacterium (*Sulfurospirillum barnesii*) postulated to be biosynthesized by a nonribosomal peptide synthetase (NRPS) polyketide synthases (PKS) hybrid. Containing a vinylogous arginine moiety, the lipodipeptide exhibited nanomolar inhibitory activity against cysteine proteases. While a putative NRPS–PKS hybrid biosynthetic gene cluster (*brn*) was identified and a *trans*‐acting acyltransferase (*trans*‐AT) domain was postulated, experimental validation remained an open question. Here, we report the production of barnesin A by heterologous expression of the *trans*‐AT domain‐dependent NRPS–PKS gene cluster in *Escherichia coli*. Our findings indicate that the native primary metabolism‐derived malonyl CoA‐acyl carrier protein transacylase homolog (FabD) functions as a *trans*‐AT in the biosynthesis pathway, while the NRPS–PKS megaenzyme exhibited strict selectivity toward its native phosphopantetheinyl transferase. Metabolome mining further allowed for the description of previously unreported barnesin congeners. The results of this study enabled the establishment of a biosynthetic platform for the generation of novel lipopeptidic vinylogous protease inhibitors.

## Graphical abstract

Vinylogous (vin) amino acids are rare and highly reactive building blocks of peptidic natural products. We report the heterologous production of the vin‐arginine harboring barnesin A, a potent cysteine protease inhibitor. Barnesin A biosynthesis is suggested to involve FabD from primary metabolism, and the corresponding NRPS–PKS megaenzyme shows strict selectivity towards the native phosphopantetheinyl transferase.

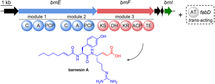

## 1 Introduction

Natural products (NPs) are structurally complex molecules with diverse biological activities and constitute a major source of novel therapeutic agents [1]. Many NPs are biosynthesized by megasynthetases, such as nonribosomal peptide synthetases (NRPSs) and polyketide synthases (PKSs), which are modular, multifunctional enzymes in which each module incorporates a specific building block in an assembly‐line manner [2]. In recent years, more and more examples of hybrid NRPS and PKS assemblies have been described, with the architectural arrangement of PKS and NRPS modules determining the resulting hybrid structural scaffold [3, 4]. While in PKS–NRPS hybrids, an upstream PKS extends the growing polyketide chain prior to amino acid incorporation by the NRPS, the downstream PKS unit in NRPS–PKS hybrids introduces and modifies the β‐keto amino acid intermediate. In most cases, NRPS–PKS assembly lines enable the homologation of proteogenic amino acids, ultimately yielding γ‐peptide motifs. For the formation of γ‐amino acids containing α,β‐unsaturated double bonds, so called vinylogous (vin) amino acids, partially reducing PKS modules consisting of a ketosynthase (KS), acyltransferase (AT), ketoreductase (KR), and dehydratase (DH) domain are required downstream of an NRPS. Additionally, *trans*‐AT systems rely on discrete, free‐standing *trans*‐acting AT‐enzymes that are typically encoded in close genomic proximity to the PKS genes [5, 6].

**SCHEME 1:**
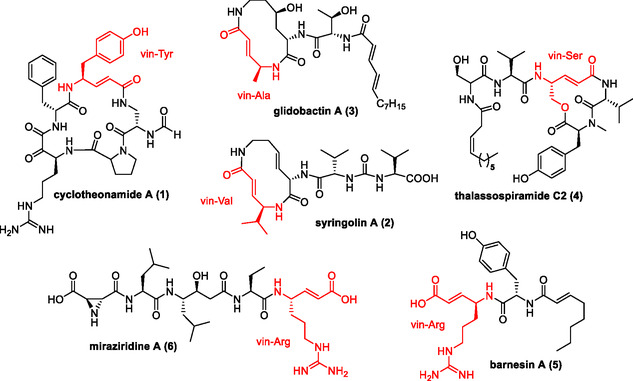
Chemical structures of natural products containing vinylogous amino acid moieties.

The electrophilic nature of the α,β‐unsaturated amide moiety (molecular warhead) activates these NPs for conjugate addition reactions, a key reactivity of protease inhibitors [7]. NPs containing such highly reactive vinylogous amino acid moieties are rare, and the most prominent examples are cyclotheonamide, syringolines [8], and glidobactins (collectively termed the syrbactins) [9, 10, 11]. The macrocyclic cyclotheonamide A (**1**) was isolated from the marine sponge *Theonella* sp. and contains structurally unique vin‐tyrosine moiety (Scheme 1). It exhibits activity against serine proteases, such as human trypsin and human two‐chain tissue plasminogen activator within the nanomolar range. Syringolins, such as syringolin A (**2**) [12], are produced by the plant pathogen *Pseudomonas syringae*, whereas glidobactins, including glidobactin A (**3**), originate from select *Burkholderiales* and *Enterobacterales* species [13, 14]. These compounds exhibit potent proteasome inhibition activity [15] and bind covalently to the β5 subunit, as confirmed by crystal structures with the yeast proteasome. In particular, promising anticancer activity in preclinical models motivated the design and evaluation of novel glidobactin analogs with improved therapeutic potential [16]. Similarly, thalassospiramide C2 (**4**), derived from marine α‐proteobacterium *Thalassospira*, bears an unusual homologated serine moiety and shows immunosuppressive activity in an interleukin‐5 production inhibition assay [17, 18]. More recently, barnesin A (**5**), a lipodipeptide containing a rare vin‐arginine moiety, was the first hybrid secondary metabolite reported from the bacterial class Campylobacteria (*Sulfurospirillum barnesii*) [19, 20] and also the first metabolite reported from anaerobic bacteria to be biosynthesized by a NRPS–PKS hybrid [21, 22, 23]. Bioactivity studies of **5** and synthetic derivatives revealed selective nanomolar inhibitory activity against the cysteine proteases cathepsin L (*K*_i_ = 1.5 µM), a proposed biomarker in cancer, and rhodesain (*K*_i_ = 81 nM), a central enzyme in the life cycle of *Trypanosoma brucei*—the causative agent of African sleeping sickness [24, 25]. The inhibition mechanism of barnesin A (**5**) was indicated to be via a Michael addition reaction to the enzymatic target. To date, only one additional NP bearing a vin‐arginine moiety, miraziridine A (**6**), has been reported, which was isolated in 2000 [26, 27].

While the hybrid biosynthetic gene clusters responsible for the production of cyclic peptides such as thalassospiramides (*ttc/ttt*), syringolines (*syl*), and glidobactins (*glb*) [9] have been studied in the past, the biosynthetic origin of miraziridine A has remained elusive [28]. For barnesin A (**5**) a putative NRPS–PKS hybrid BGC, designated *brn*, was only tentatively assigned. Given the extremely low native production levels (0.014 mg L^−1^) of barnesin A (**5**), the complexity of its multistep chemical synthesis, and its promising bioactivity, biosynthetic engineering offers an attractive alternative for scalable production and potential structural diversification in a reproducible and sustainable manner. Thus, we pursued heterologous expression of the *brn* biosynthetic gene cluster as an initial step to validate the biosynthetic origin and enable the establishment of a biosynthetic platform for the generation of novel lipopeptidic vinylogous protease inhibitors.

## 2 Results and Discussion

### 2.1 Analysis of the *brn* Gene Cluster

**FIGURE 1:**
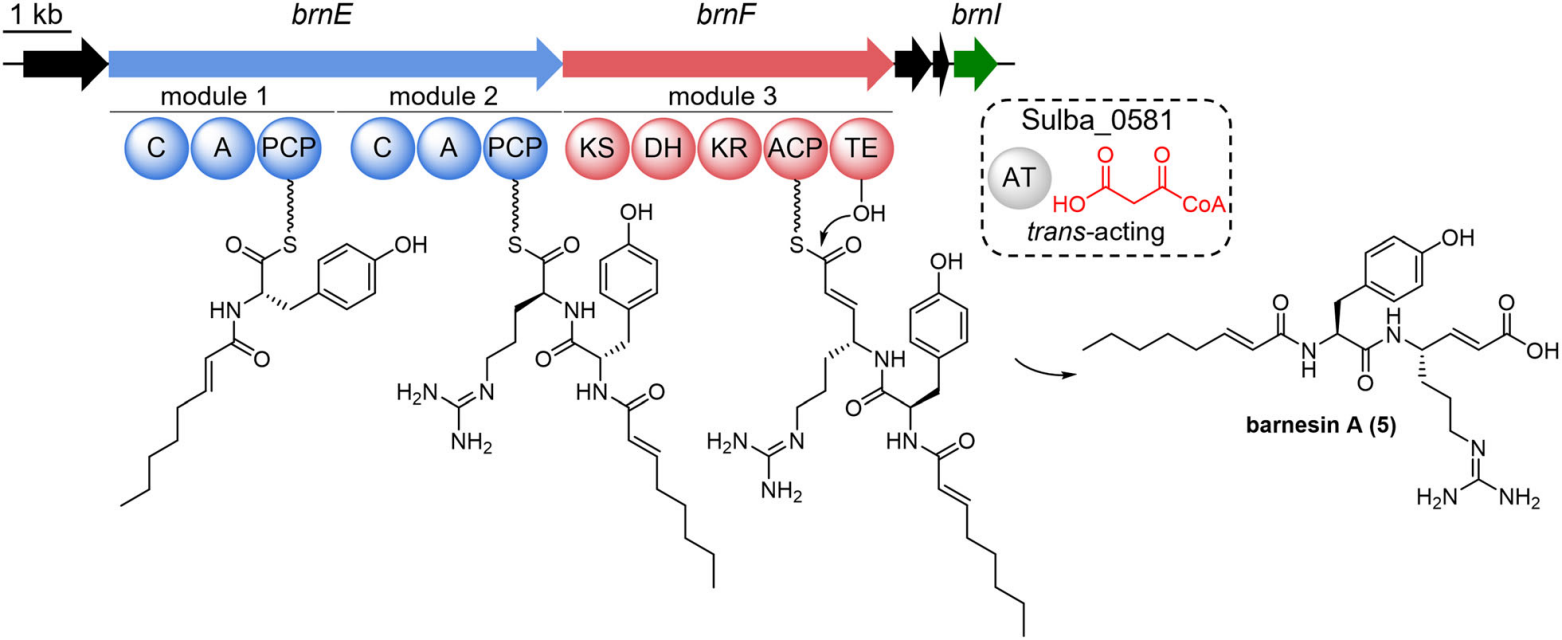
Architecture of the *brn* BGC and putative biosynthesis of barnesin A (**5**); *brnE* (NRPS), *brnF* (PKS), and *brnI* (PPTase) are encoded within the set boundaries of the BGC. The acyltransferase (AT) Sulba_0581 required for providing the malonyl‐CoA extender unit is not encoded within the BGC. Domain abbreviations: condensation (C), adenylation (A), peptidyl carrier protein (PCP), ketosynthase (KS), dehydratase (DH), ketoreductase (KR), acyl carrier protein (ACP), thioesterase (TE).

The *brn* cluster from *S. barnesii* and its homologous sequences *gvb* from *Geovibrio* sp. L21‐Ace‐BES are approximately 20 kbp in size and encode for an NRPS (*brn*E), a PKS (*brn*F), a cyclic peptide transporter (*brn*BCD), and a phosphopantetheinyl transferase (PPTase) (*brn* *I*) (Figure 1) [19]. To this day, no additional homologous BGC could be identified by dedicated BLAST queries. The *trans*‐AT PKS BrnF comprises a KS‐, KR‐, and DH‐domain necessary for the production of a vin‐amino acid as well as a thioesterase (TE) domain for chain release of the mature biosynthetic product. Specificity analyses of the biosynthetic domains in both clusters revealed that the first condensation (C) domain of BrnE functions as a starter C‐domain and is likely responsible for fatty acid transfer during lipopeptide formation, whereas the second C‐domain catalyzes condensation between two *L*‐amino acids. The first adenylation (A) domain of BrnE belongs to a previously unrecognized subgroup within this class and was predicted to activate aromatic amino acids, while the second A‐domain exhibits a binding pocket consistent with activation of arginine or lysine residues. Employing the recently released PARAS tool [29], both tyrosine and arginine were predicted as the respective substrates, as observed within the mature lipopeptide.

Lacking an intrinsic AT‐domain, the *trans*‐AT PKS BrnF is dependent on a *trans*‐acting acyltransferase to provide the malonyl extender unit required for the introduction of the vinyl group. In prior work, *sulba_0581* (WP_0147688770.1), annotated as the malonyl CoA‐acyl carrier protein transacylase FabD, was identified as the most likely *trans*‐AT gene candidate. However, its validation remained an open question. FabD enzymes usually provide malonyl extender units during Type II fatty acid biosynthesis [30]. While it is not unprecedented that FabD can perform *trans*‐AT function in both fatty acid and PKS biosynthesis, the few described examples mainly occurred in Type II PKS systems from *Streptomyces.* [31, 32]. Only recently, FabD was also shown to be involved in the biosynthesis of pseudotetraivprolide and detoxins, produced by *Pseudomonas* strains [33].

**FIGURE 2:**
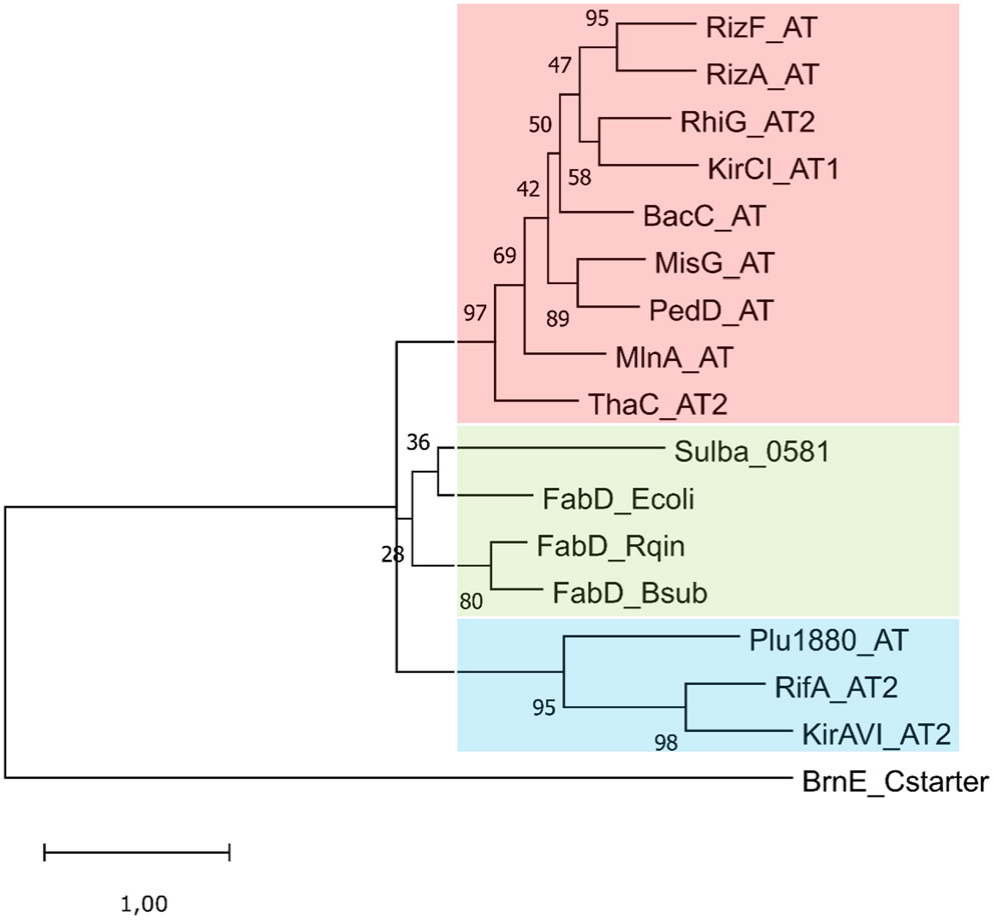
Phylogenetic tree of Sulba_0581 with *cis*‐AT, *trans*‐AT, and FabD reference sequences. Maximum likelihood tree inferred using 1000 bootstrap repetitions. Highlighted in red are *trans*‐AT enzymes involved in PKS biosynthesis, in green *trans*‐AT enzymes involved in fatty acid biosynthesis, and in blue *cis*‐AT enzymes involved PKS biosynthesis. BrnE_Cstarter serves as the outgroup.

Thus, in a first step, we re‐evaluated the identity of the *trans*‐AT working candidate Sulba_0581 to provide the basis for subsequent heterologous expression. For this, the *S. barnesii* SES‐3 (CP003333.1) genome was again mined for the presence of *trans*‐acting acyltransferases by BLASTp searches using literature‐known *trans*‐AT and *cis*‐AT protein sequences. To prevent a taxonomic bias in the analysis, query sequences from taxonomically diverse hosts were selected (Table S4). Almost all searches resulted in the exclusive reidentification of Sulba_0581 (Table S5). The BLASTp results were further corroborated by a phylogenetic tree, in which Sulba_0581 clustered into a monophyletic clade with FabD variants of *Escherichia coli* K‐12 MG1655, *Bacillus subtilis* strain 168, and *Rhodococcus qingshengii* S‐E5 (Figure 2). This clustering suggests that Sulba_0581 may serve a hybrid role in both primary and specialized secondary metabolism [33].

### 2.2 Heterologous Expression of the *brn* BGC

To evaluate the feasibility of heterologous barnesin A (**5**) production, we first examined whether the individual biosynthetic enzymes could be expressed in a soluble form in *E. coli*. As the identity of the N‐terminal amino acid residue is a key determinant of protein stability in the cytosol and nucleus (N‐end rule), proteins bearing arginine, leucine, lysine, phenylalanine, tryptophan, or tyrosine at the N‐terminus exhibit markedly reduced in vivo half‐lives of approximately 2 min. In contrast, proteins with alternative N‐terminal residues display substantially prolonged half‐lives, often exceeding several hours [34, 35]. To assess the stability and estimate relative protein half‐lives of BrnE, BrnF, BrnI, and Sulba_0581, the N‐terminal amino acid residues of the four relevant biosynthetic enzymes were manually curated and analyzed using Geneious Prime (v2022.2.2). This analysis revealed that both BrnE (detection of leucine) and BrnF (detection of lysine and leucine) possess destabilizing N‐terminal residues (Figure S1). To ensure high and sustainable protein concentrations during heterologous expression experiments, the codons encoding for the destabilizing N‐terminal amino acid residues were removed from the respective gene sequences during construction of the expression plasmids.

The corresponding modified synthetic genes were then individually cloned into expression vectors under the control of an IPTG‐inducible T7 promoter. Using *E. coli* BL21(DE3) as expression host, the smaller genes *sulba_0581* and *brnI* were readily expressed, with protein production confirmed by SDS–PAGE analysis (Figure S2). The larger PKS‐encoding gene *brnF* was also successfully expressed; however, detectable protein production required extended cultivation for 3 days at 18°C. In contrast, production of the NRPS BrnE could not be confirmed, neither with reduced expression temperatures nor prolonged cultivation times.

We then performed coexpression of *sulba_0581*, *brnE*, and *brnF* to assess whether the coproduction would allow for sufficient protein levels to support barnesin A (**5**) production. For this purpose, *E. coli* BL21(DE3) derivative BAP1 was selected as the expression host [36], as it already encodes the broad range PPTase Sfp from *B. subtilis*, necessary for activation of the hybrid assembly line. However, also coexpression experiments remained unsuccessful despite investigations of different cultivation conditions and supplementation of precursor fatty acid, leaving barnesin A (**5**) nondetectable.

Given the substantial taxonomic distance between the native producer *S. barnesii* SES‐3 (phylum Campylobacterota) and the heterologous host *E. coli* BL21(DE3) (phylum Pseudomonadota), differences in codon usage and tRNA availability were considered as potential limiting factors. However, even a codon‐optimized *brnE_mcu* gene sequence, using the match codon usage (mcu) algorithm [37], failed to produce detectable protein amounts of BrnE_mcu. We then analyzed whether promoter strength and regulation critically influenced heterologous protein production. In particular, the strong T7 promoter is prone to leaky expression and imposes a substantial metabolic burden on the host, often resulting in low protein yields.

To enable tighter transcriptional control, expression constructs driven by the *ara*BAD promoter were generated. Because *E. coli* BL21(DE3) metabolizes the inducer *L*‐arabinose, the expression host was switched to *E. coli* DH10B::*mtaA*, which carries the *araD139* mutation that prevents *L*‐arabinose catabolism [38]. Additionally, it has *mta*A, encoding for a broad range PPTase from *Stigmatella aurantiaca*, integrated into its genome. Using the new expression system, soluble production of BrnE_mcu was finally achieved and *brnF_mcu* expression was again verified (Figure S3). Consequently, *brnE_mcu*, *brnF_mcu*, and *sulba_0581* were coexpressed in *E. coli* DH10B::*mtaA*. Since production of *trans*‐2‐octenoic acid has so far not been described in *E. coli*, the monounsaturated fatty acid was supplemented during heterologous production experiments. Despite detectable protein levels by SDS–PAGE, HRMS‐based metabolomic analyses of SPE‐enriched extract failed to detect barnesin A (**5**) or derivatives incorporating saturated fatty acid moieties. At this stage, it is important to note that we also evaluated *Pseudomonas protegens* Pf‐5 Δ*gacA* (phylum: Pseudomonadota) as an alternative expression host as it possesses a metabolism inherently more adapted to NP biosynthesis, including several *trans*‐AT PKSs, than that of *E. coli* [39]. However, neither coexpression of *brnE*‐*brnF* nor the *E. coli*‐codon optimized variants *brnE_mcu*‐*brnF_mcu* resulted in detectable protein production or metabolite production in the presence of *trans*‐2‐octenonic acid (Figure S4).

### 2.3 Native PPTase BrnI Is Essential to Render the PKS–RNPS Hybrid Active

**FIGURE 3:**
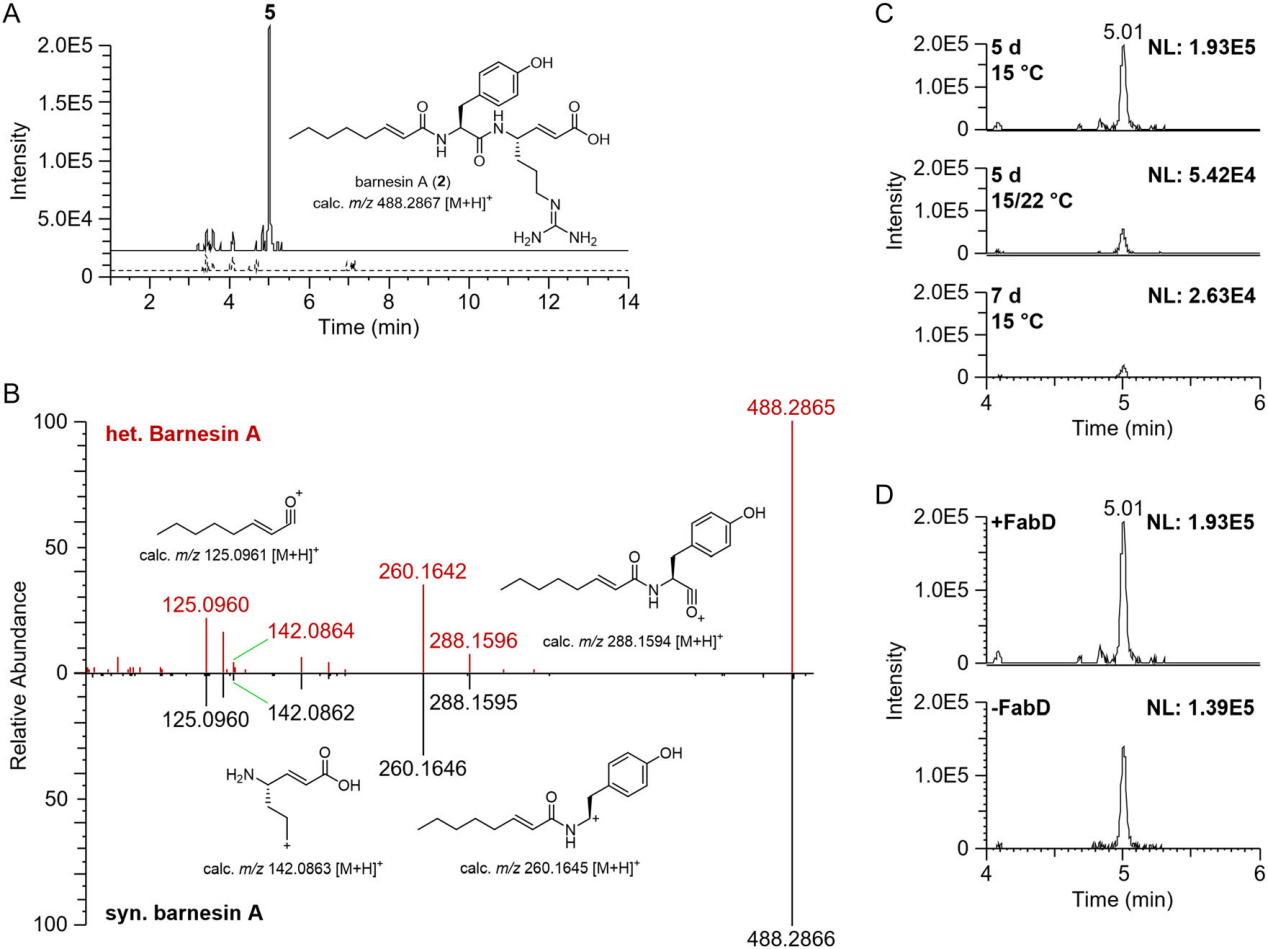
Heterologous production of barnesin A. (A) EIC (**5**, *m/z* 488.2867 [M+H]^+^) comparison of *brn* expression construct (solid line) and no plasmid control (dashed line). (B) MS^2^ spectra comparison of **5** (top‐red) and synthetic barnesin A (bottom‐black). (C) EIC (**5**, *m/z* 488.2867 [M+H]^+^) comparison of multiple expression conditions (top = 5 days at 15°C; middle = 3 days at 15°C followed by 2 days at 22°C; bottom = 7 days at 15°C). (D) EIC (**5**, *m/z* 488.2867 [M+H]^+^) comparison of expression conditions including *sulba_0581* (+FabD = *brnE_mcu*, *brnF_mcu*, *brnI*, *sulba_0581*; top) and conditions lacking *sulba_0581* (‐FabD = *brnE_mcu*, *brnF_mcu*, *brnI*; bottom). NL = normalization level.

At this stage, we speculated that the broad‐range PPTase MtaA does not accept BrnE_mcu and BrnF_mcu as substrates, and both were still present in their inactive *apo*‐form. To test this hypothesis, *brnI* (native PPTase) was coexpressed alongside *brnE_mcu*, *brnF_mcu*, and *sulba_0581* in *E. coli* DH10B, while simultaneously reducing the expression temperature to 15°C and elongating the cultivation time to 5 days. Under these conditions, a new mass feature (*m/z* 488.2869 [M+H]^+^, RT = 5.01 min) with identical *m/z* as **5** was identified. Indeed, comparison of retention times and MS^2^ fragmentation patterns with internally available synthetic barnesin A (**5**) confirmed the mass feature, thereby validating for the first time the *brn* BGC as the biosynthetic origin of barnesin A (Figures 3A,B and S5). To assess the impact of *trans*‐2‐octenoic acid supplementation on production titers, heterologous expression experiments were comparatively analyzed in the presence and absence of the monounsaturated fatty acid. Notably, omission of *trans*‐2‐octenoic acid did not abolish barnesin production (Figure S6). This observation suggests the presence of ACP‐bound 2‐octenoic acid in *E. coli*, most likely representing intermediates of the endogenous Type II fatty acid biosynthetic pathway. We also evaluated the effect of time and temperature on the production of **5**; however, among the conditions tested, cultivation time of 5 days at 15°C yielded the most reliable production levels (Figures 3C and S7). To investigate the influence of the *trans*‐AT Sulba_0581 on barnesin biosynthesis, coexpression experiments were performed either including or excluding the putative *trans*‐acting acyltransferase of *S. barnesii*. Strikingly, the omission of Sulba_0581 resulted in only a slight reduction in barnesin production, which indicates that an endogenous *E. coli* homolog is likely able to complement the *trans*‐acting acyltransferase activity required for barnesin biosynthesis (Figures 3D and S8).

**FIGURE 4:**
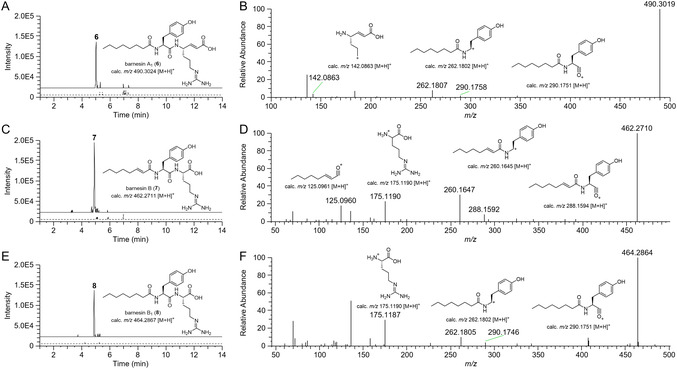
Metabolome mining for barnesin congeners. (A) EIC (**6**, *m/z* 490.3024 [M+H]^+^) of *brn* expression construct (solid line) in comparison to no plasmid control (dashed line). (B) MS^2^ spectra of barnesin A_1_ (**6**). (C) EIC (**7**, *m/z* 462.2711 [M+H]^+^) of *brn* expression construct (solid line) in comparison to no plasmid control (dashed line). (D) MS^2^ spectra of barnesin B (**7**). (E) EIC (**8**, *m/z* 464.2867 [M+H]^+^) of *brn* expression construct (solid line) in comparison to no plasmid control (dashed line). (F) MS^2^ spectra of barnesin B_1_ (**8**).

Due to the ambiguity related to the ability to activate unsaturated fatty acid and the specificity of the A‐domain, we also analyzed the metabolome for possible fatty acid as well as amino acid congeners. Here, we speculated that the first A‐domain of BrnE could show some promiscuity toward either phenylalanine, leucine, or valine, while the second was hypothesized to possibly accept histidine, asparagine, glutamine, or lysine based on their diagnostic A‐domain signatures [40]. Targeted metabolome mining for the theoretical mass features, however, only resulted in the identification of barnesin congeners carrying tyrosine and arginine moieties. While focusing on congeners differing in their fatty acid moiety, the analysis resulted in the identification of mass features **6** (*m/z* 490.3024 [M+H]^+^, RT = 5.01 min), **7** (*m/z* 462.2711 [M+H]^+^, RT = 4.91 min), and **8** (*m/z* 464.2867 [M+H]^+^, RT = 4.91 min) (Figure 4). Having a mass difference of +2 Da relative to **5** as well as the same retention time, **6** was assumed to carry octanoic acid instead of the mono unsaturated fatty *trans*‐2‐octenonic acid. MS^2^ analysis confirmed the incorporation of the saturated fatty acid, and consequently, **6** was termed barnesin A_1_ [19, 24]. In comparison, **7** displayed a mass difference of −26 Da to **5**, indicating the loss of a C_2_H_2_ unit. Careful, MS^2^‐based fragment analysis, however, revealed **7** to be a biosynthetic shunt metabolite retaining *trans*‐2‐octenonic acid and incorporating an arginine instead of the vin‐arginine; hence, **7** was termed barnesin B. Similar to **5** and **6**, **8** exhibited a mass difference of +2 Da relative to **7** while sharing the same retention time. Based on the MS^2^ analysis, **8** was assigned as the octanoic acid congener and thus termed barnesin B_1_.

## 3 Conclusion

In this study, we validated the *brn* biosynthetic pathway through heterologous expression in *E. coli*. Functional reconstitution required codon optimization of the NRPS gene sequence as well as expression of the native PPTase *brnI*, ultimately enabling the formation of active enzyme complexes capable of synthesizing barnesin A (**5**). Our findings also indicated that FabD functions as the *trans*‐AT and serves a hybrid role in both primary and specialized secondary metabolism in the native host. Metabolomic analyses further revealed a degree of substrate promiscuity toward related fatty acid chains, resulting in the production of barnesin congeners (**6**, **7**, and **8**).

## Supporting Information

Additional supporting information can be found online in the Supporting Information section. Experiment details and analytical data are available within the Supporting Information. The authors have cited additional references within the Supporting Information.

## Funding

This study was supported by Horizon 2020 Framework Programme (802736).

## Conflicts of Interest

The authors declare no conflicts of interest.

## Supporting information

Supplementary Material

## Data Availability

Supplementary Information contains all details to experimental and analytical data. The MS data was uploaded to the MAssIVE Server (MSV000100654).
