# Supplementary material for: Heterologous Production of Barnesin A, an NRPS–PKS Hybrid Containing a Rare Vinylogous Arginine Moiety
Source: Chembiochem. 2026 Apr 21;27(8):e70305. doi: 10.1002/cbic.70305 (PMC13096860; doi:10.1002/cbic.70305)
Supplement: Supplementary file 1 — Supplementary Material [file CBIC-27-e70305-s001.pdf]

Supporting Information

Heterologous Production of Barnesin A, a NRPS-PKS Hybrid Containing a Rare Vinylogous Arginine Moiety

Sven Balluff,[a] Marie Dayras,[a] Christine Beemelmanns[a,b]*

Table of Contents

Table of Contents 1

Experimental Procedures 2
Validation of *trans*-AT working candidate Sulba_0581 2
Construction of expression plasmids 2
Heterologous expression 2
SDS–PAGE Analysis 3
Culture Extraction 4
UHPLC-HRMS Analysis 4

Supplementary Tables 5
Table S1. Organisms used in heterologous expression experiments of the *brn* BGC. 5
Table S2. Plasmids used in heterologous expression experiments of the *brn* BGC. 6
Table S3. Oligonucleotides used for the construction of *brn* expression plasmids. 7
Table S4. Protein sequences used for validation *trans*-AT working candidate Sulba_0581. 9
Table S5. BLASTp search of selected *trans*- and *cis*-AT query sequences against the *S. barnesii* SES-3 genome. 9

Supplementary Figures 10
Figure S1. N-terminal amino acid residues of the four biosynthetic enzymes required for barnesin A production 10
Figure S2. SDS–PAGE analyses of heterologous expression experiments using *E. coli* BL21(DE3). 10
Figure S3. SDS–PAGE analyses of heterologous expression experiments in *E. coli* DH10B. 11
Figure S4. SDS–PAGE analyses of heterologous expression experiments in *P. protegens* Pf-5 Δ*gacA*. 11
Figure S5. Retention time comparison. 12
Figure S7. Efforts to increase barnesin production. 13
Figure S8. Influence of *sulba_0581* expression on barnesin biosynthesis. 13

Supplementary References 14

Experimental Procedures

Validation of *trans*-AT working candidate Sulba_0581

To evaluate whether Sulba_0581 represents the most likely *trans*-AT candidate involved in barnesin A biosynthesis, randomly selected *trans*- and *cis*-AT protein sequences from various PKS BGCs were used as queries in BLASTp searches against the *Sulfurospirillum barnesii* SES-3 genome (GenBank: CP003333.1). The top hits from these searches were further aligned with the previous mentioned and additional *trans*-AT protein sequences from the fatty acid biosynthesis using the MUSCLE algorithm. A phylogenetic tree was subsequently constructed in MEGA11 using the maximum likelihood method with the LG+G substitution model and 1,000 bootstrap replicates.[1] The protein sequence from the BrnE $C_{starter}$-domain was used as the outgroup.

Construction of expression plasmids

Expression plasmids were generated using the NEBuilder® HiFi DNA Assembly Cloning Kit (New England Biolabs) and were verified by colony PCR as well as Sanger Sequencing (Eurofins Genomics). Final expression constructs were transformed into electrocompetent *E. coli* BL21(DE3), *E. coli* DH10B, *E. coli* DH10B::*mtaA* or *P. protegens* Pf-5 Δ*gacA* cells using standard electroporation protocols.[2,3]

Heterologous expression

Heterologous expression experiments in *E. coli* BL21(DE3) or BAP1,[4] using the isopropyl β-*D*-1-thiogalactopyranoside (IPTG) inducible T7 operon, were conducted in 20 mL cultures of using lysogenic broth (LB) media with appropriate antibiotics (100 µg/mL ampicillin, 50 µg/mL apramycin or 50 µg/mL kanamycin). Cultures were inoculated with 100 µL of a well grown pre-culture and cultivated at 37 °C with shaking at 180 rpm until an OD_{600} of 0.6-0.8 was reached. Prior to induction, cultures were cooled on ice for 15 min. Protein expression was then induced by addition of IPTG to a final concentration of 0.1 mM. Following induction, cultures were incubated for up to 3 d at 18 °C with shaking at 180 rpm. 2 mM *trans*-2-octeonic acid was supplemented during induction, after 24 h and after 48 h.

Heterologous expression experiments in *E. coli* DH10B or *E. coli* DH10B::*mtaA,* using the *L*-arabinose inducible *araBAD* operon, were conducted in 20 mL cultures using either LB or XPP media with appropriate antibiotics (50 µg/mL apramycin, 34 µg/mL chloramphenicol or 50 µg/mL kanamycin).[5,6] Cultures were inoculated with 100 µL of a well grown pre-culture and protein expression was induced at the beginning of the cultivation by adding *L*-arabinose to a final concentration of 0.02 % (w/v). Cultures were incubated either for 3 d at 18 °C or for 5 d at 15 °C, with shaking at 180 rpm. 1 mM *trans*-2-octeonic acid was supplemented during the 5 d

cultivations after 6 h, 24 h, 48 h, 72 h and 96 h. To enhance barnesin A production, two approaches were undertaken. In the first approach, the heterologous expression experiment was initially carried out for 3 d at 15 °C followed by another cultivation step for 2 d at 22 °C with 2 mM *trans*-2-octeonic acid supplementation after 6 h, 24 h, 48 h, 72 h, 96 h. In the second approach, the heterologous expression experiment was elongated to 7 d at 15 °C with 2 mM *trans*-2-octeonic acid supplementation after 6 h, 24 h, 48 h, 72 h, 96 h, 120 h and 144 h.

Heterologous expression experiments in *P. protegens* Pf-5 Δ*gacA*, using the *L*-arabinose inducible *araBAD* operon, were conducted in 20 mL cultures using either LB or XPP media with appropriate antibiotics (50 µg/mL apramycin).[6] Cultures were inoculated with 100 µL of a well grown pre-culture and protein expression was induced at the beginning of the cultivation by adding *L*-arabinose to a final concentration of 0.2 % (w/v). Cultures were then incubated for 24 h at 18 °C with shaking at 180 rpm. 2 mM *trans*-2-octeonic acid was supplemented during induction, after 6 h and 12 h.

SDS–PAGE Analysis

Protein production was monitored using sodium dodecyl sulfate-polyacrylamide gel electrophoresis (SDS–PAGE). Therefore, cells were harvested by centrifugation at 12,000 × g and 4 °C for 10 min. The resulting cell pellet was resuspended in 6 mL lysis buffer (250 mM NaCl, 100 mM HEPES, 1 mM EDTA, 0.1 % [v/v] Triton X-100, pH 7.6) supplemented with 60 µL protease inhibitor cocktail (Sigma-Aldrich). The bacterial suspension was cooled on ice and all subsequent steps were carried out on ice as well. Cell lysis was achieved by sonication for 10 min using a MS73 sonotrode (Bandelin) at 40 % amplitude with a 2 s on/3 s off pulse cycle. To enhance lysis efficiency, a spatula tip of lysozyme (Sigma-Aldrich) was added to the suspension 10 min prior to sonication. Following lysis, the suspension was centrifuged for 20 min at 12,000 × g and 4 °C. The supernatants were collected as the soluble fractions and the remaining cell debris pellets were resuspended in 6 mL diH_2O and designated as the pellet fractions. Protein concentrations in each fraction were determined spectrophotometrically using the EzDrop 1000 (Blue-Ray Biotech). Samples were then adjusted to a final concentration of 50 µg protein in 12.5 µL Laemmli buffer (Bio-Rad). Prior to electrophoresis, samples were denatured by heating at 95 °C for 10 min. Proteins were separated on 4–15 % Mini-PROTEAN® TGX™ or Any kD Mini-PROTEAN® TGX™ precast protein gels (Bio-Rad) and electrophoresis was conducted for 10 min at 60 V, followed by for 60 min at 100 V. Gels were stained by immersing in excess staining solution (70 mg/L Coomassie G250, 3 mL/L 37 % HCl) microwaving for 20 s at 600 W and incubating under constant shaking for 20 min. The process was repeated twice, with the final incubation period conducted overnight. To reduce background staining, gels were washed in excess diH_2O, microwaved for 40 s at 600 W and

incubated under shaking for 20 min. The washing procedure was repeated twice, with the final incubation period extended until sufficient background reduction was achieved. For documentation a GelStick IMAGER (Intas) was used.

Culture Extraction

For metabolomic analysis of heterologous expression cultivations, the cultures were harvested by centrifugation for 10 min at 10,000 × g. Metabolites were extracted from the resulting supernatants via solid-phase extraction (SPE) using CHROMABOND® 500 mg/3 mL C18ec SPE columns (Macherey-Nagel). Therefore, the supernatants were adjusted to a final concentration of 10 % MeOH and processed according to the following SPE protocol: (1) column activation with 3 CV MeOH (2) column equilibration with 3 CV 10 % MeOH (3) sample loading (4) washing with 3 CV 10 % MeOH (5) elution with 3 CV 50 % MeOH, 3 CV 80 % MeOH and 3 CV MeOH. Additionally, the cell pellets were extracted for 1 h in 10 mL MeOH and combined with the SPE elution fractions. The combined fractions were then dried under reduced pressure.

UHPLC-HRMS Analysis

UHPLC-HRMS measurements were carried out on a Vanquish Flex UHPLC system (Thermo Fisher Scientific) combined with an Orbitrap Exploris 120 mass spectrometer equipped with a HESI source (Thermo Fisher Scientific). Metabolites were separated using reverse phase liquid chromatography at 40 °C using a Kinetex® C18 column (50 × 2.1 mm, particle size 1.7 µm, 100 Å, Phenomenex) preceded by a C18 SecurityGuardTM ULTRA guard cartridge (2.1 mm, Phenomenex). Mobile phases consisted of H_2O + 0.1 % formic acid (A) and acetonitrile (ACN) + 0.1 % formic acid (B). 5 µL sample, concentrated at 50 µg/mL, was injected into a gradient as follows: 0-1 min, 5 % B; 1-10 min, 5-97 % B; 10-12 min, 97 % B; 12-13 min, 97-5 % B; 13-15 min, 5 % B at a constant flow rate of 0.3 mL/min. Data dependent acquisition of MS^2 spectra was performed in positive mode. HESI parameters were set to 50 AU sheath gas flow, 13 AU auxiliary gas flow, 1 AU sweep gas flow, 3.4 kV (+) spray voltage, 300 °C vaporizer temperature and 320 °C ion transfer tube temperature. MS^1 full scan parameters were set to data type-centroid, *m/z* 150-1,500 scan range, resolving power 60,000 at *m/z* 200, 1 micro-scan, 100 ms max. injection time, 1E6 automated gain control, 70 % RF lens, dynamic exclusion filter-auto and isotope exclusion filter-assigned. Up to 4 MS^2 spectra per MS^1 survey scan were recorded with the following parameters, data type-centroid, scan range-auto, resolving power 30,000 at *m/z* 200, 1 micro-scan, 100 ms max. injection time, 1E5 automated gain control, 1.2 *m/z* isolation window, collision energy type- normalized with a stepwise increase from 20 to 30 to 40 %.

Supplementary Tables

Table S1. Organisms used in heterologous expression experiments of the *brn* BGC.

Strain	Genotype	Reference
Escherichia coli BL21(DE3)	F^-*ompT hsdS*$_B$ (r_B–, m_B–) *gal dcm* (DE3)	Thermo Fisher Scientific
Escherichia coli BAP1	F^-*ompT hsdS*$_B$ (r_B–, m_B–) *gal dcm* (DE3) Δ*prp*RBCD::T7prom-*sfp* T7prom-*prpE*	4
Escherichia coli DH10B	F^-*mcr*A Δ*(mrr-hsd*RMS-*mcr*BC*)* Φ80*lac*ZΔM15 Δ*lac*X74 *rec*A1 *end*A1 *ara*D139 Δ(*ara-leu*)7697 *gal*U *gal*K λ^- *rps*L(Str^R) *nup*G	Thermo Fisher Scientific
Escherichia coli DH10B::*mtaA*	F^-*mcr*A Δ*(mrr-hsd*RMS-*mcr*BC*)* Φ80*lac*ZΔM15 Δ*lac*X74 *rec*A1 *end*A1 *ara*D139 Δ(*ara-leu*)7697 *gal*U *gal*K λ^- *rps*L(Str^R) *nup*G *endD*::*mtaA*	[5]
Pseudomonas protegens Pf-5 Δ*gacA*	Δ*gacA*	[7]

Table S2. Plasmids used in heterologous expression experiments of the *brn* BGC. Plasmids pSB23 and pSB32 were commercially obtained from BioCat.

Name	Description	Size	Reference
pET28a(+)	pBR322 ori, f1 ori, *kan*^{R}, T7 promoter, T7 terminator	5,365 bp	Merck Millipore
pETDuet-1	pBR322 ori, f1 ori, *amp*^{R}, T7 promoter, T7 terminator	5,419 bp	Merck Millipore
pCDFDuet-1Apr	CDF ori, *spt*^{R}, *spr*^{R}, *apr*^{R}, T7 promoter, T7 terminator	4,764 bp	Beemelmanns group
pJW75	p15A ori, *cam*^{R}, *araBAD* promoter, *gxpS* (A1-C3+SZ17), *araE*, T7 terminator	11,928 bp	[8]
pJW76	ColA ori, *kan*^{R}, *araBAD* promoter, *gxpS* (SZ18+A3-TE), T7 terminator	12,471 bp	[8]
pRANGER-MK3	pBBR1 ori, *apr*^{R}, *araBAD* promoter, soxR terminator	3,704 bp	[7]
pSB06	pBR322 ori, f1 ori, *kan*^{R}, T7 promoter, *sulba_0581*, T7 terminator	6,126 bp	This work
pSB07	pBR322 ori, f1 ori, *kan*^{R}, T7 promoter, *brnI*, T7 terminator	5,838 bp	This work
pSB10	pBR322 ori, f1 ori, *kan*^{R}, T7 promoter, *brnF_noK2-L5*, T7 terminator	10,023 bp	This work
pSB11	pBR322 ori, f1 ori, *kan*^{R}, T7 promoter, *brnE_noL2*, T7 terminator	11,805 bp	This work
pSB15	CDF ori, *spt*^{R}, *spr*^{R}, *apr*^{R}, T7 promoter, *brnE_noL2*, T7 promoter, T7 terminator	11,264 bp	This work
pSB16	pBR322 ori, f1 ori, *amp*^{R}, T7 promoter, *sulba_0581*, T7 promoter, T7 terminator	6,240 bp	This work
pSB18	CDF ori, *spt*^{R}, *spr*^{R}, *apr*^{R}, T7 promoter, *brnE_noL2*, T7 promoter, *brnF_noK2-L5*, T7 terminator	15,965 bp	This work
pSB23	pBR322 ori, f1 ori, *kan*^{R}, T7 promoter, *brnE_noL2_mcu*, T7 terminator	11,947 bp	This work
pSB30	p15A ori, *cam*^{R}, *araBAD* promoter, *sulba_0581*, T7 terminator	4,622 bp	This work
pSB31	p15A ori, *cam*^{R}, *araBAD* promoter, *brnE_noL2_mcu*, T7 terminator	10,301 bp	This work
pSB32	pBR322 ori, f1 ori, *kan*^{R}, T7 promoter, *brnF_noK2-L5_mcu*, T7 terminator	10,183 bp	This work
pSB34	p15A ori, *cam*^{R}, *araBAD* promoter, *brnE_noL2*, T7 terminator	10,301 bp	This work
pSB35	ColA ori, *kan*^{R}, *araBAD* promoter, *brnF_noK2-L5*, T7 terminator	7,973 bp	This work
pSB36	pBBR1 ori, *apr*^{R}, *araBAD* promoter, *brnE_noL2*, soxR terminator	10,296 bp	This work
pSB37	pBBR1 ori, *apr*^{R}, *araBAD* promoter, *brnE_noL2*, *araBAD* promoter, *brnF_noK2-L5*, soxR terminator	15,453 bp	This work
pSB40	ColA ori, *kan*^{R}, *araBAD* promoter, T7 terminator	3,155 bp	This work
pSB46	ColA ori, *kan*^{R}, *araBAD* promoter, *brnF_noK2-L5_mcu*, T7 terminator	7,973 bp	This work
pSB48	ColA ori, *kan*^{R}, *araBAD* promoter, *brnF_noK2-L5_mcu*, *brnE_noL2_mcu*, T7 terminator	14,907 bp	This work
pSB49	pBBR1 ori, *apr*^{R}, *araBAD* promoter, *brnE_noL2_mcu*, soxR terminator	10,296 bp	This work
pSB50	pBBR1 ori, *apr*^{R}, *araBAD* promoter, *brnE_noL2_mcu*, *araBAD* promoter, *brnF_noK2-L5_mcu*, soxR terminator	15,453 bp	This work
pSB51	p15A ori, *cam*^{R}, *araBAD* promoter, *brnI*, T7 terminator	4,334 bp	This work
pSB52	p15A ori, *cam*^{R}, *araBAD* promoter, *sulba_0581*, *brnI*, T7 terminator	5,590 bp	This work

Table S3. Oligonucleotides used for the construction of *brn* expression plasmids.

Name	Template	Usage	Sequence (5'-3')
SB039	pET28a(+)<br>pETDuet-1<br>pCDFDuet-1Apr	bb pSB06, pSB07, pSB10, pSB11 (rev)<br>bb pSB07 (rev)<br>bb pSB16 (rev)	CATGGTATATCTCCTTCTTAAAGTTAAAC
SB041	pET28a(+)	bb pSB06, pSB07, pSB10, pSB11 (fwd)	GATCCGGCTGCTAACAAAG
SB042	*S. barnesii* SES-3	*sulba_0581* for pSB06 (fwd)	AACTTTAAGAAGGAGATATACCATGAGTAATACAGTGTTTATATTTCCTG
SB043	*S. barnesii* SES-3	*sulba_0581* for pSB06 (rev)	TTCGGGCTTTGTTAGCAGCCGGATCTTAAAACAGTGCAAGCAC
SB044	*S. barnesii* SES-3	*brnI* for pSB07 (fwd)	AACTTTAAGAAGGAGATATACCATGCACCTTGAAACTGTCTTACTAC
SB046	*S. barnesii* SES-3	*brnI* for pSB07 (rev)	TTCGGGCTTTGTTAGCAGCCGGATCTTAATCTAAAGCCCGTAATTCG
SB047	*S. barnesii* SES-3	*brnE_noL2* for pSB11 (fwd)	GAAATAATTTTGTTTAACTTTAAGAAGGAGATATACCATGAATATATACGCACTTTCATCACC
SB049	*S. barnesii* SES-3	*brnE_noL2* for pSB11 (rev)	CCAACTCAGCTTCCTTTCGGGCTTTGTTAGCAGCCGGATCTCATGAGGCTGCCTGTATAATC
SB050	*S. barnesii* SES-3	*brnF_noK2-L5* for pSB10 (fwd)	GAAATAATTTTGTTTAACTTTAAGAAGGAGATATACCATGGACCTTAAAAATGCCATAAAGC
SB052	*S. barnesii* SES-3	*brnF_noK2-L5* for pSB10 (rev)	CCAACTCAGCTTCCTTTCGGGCTTTGTTAGCAGCCGGATCTTACTTTAATGTGTTTAAAATACT<br>TC
SB055	pCDFDuet-1Apr	bb pSB15 (rev)	CATGGTATATCTCCTTATTAAAGTTAAAC
SB059	pCDFDuet-1Apr<br>pETDuet-1	bb pSB15 (fwd)<br>bb pSB16 (fwd)	CAGAAAGTAATCGTATTGTACACG
SB060	*S. barnesii* SES-3	*brnE_noL2* for pSB15 (fwd)	TTTAATAAGGAGATATACCATGAATATATACGCACTTTCATCACC
SB061	*S. barnesii* SES-3	*brnE_noL2* for pSB15 (rev)	GTACAATACGATTACTTTCTGTCATGAGGCTGCCTGTATAATC
SB062	pSB15	bb-*brnE_noL2* for pSB18 (rev)	CATATGTATATCTCCTTCTTATAC
SB072	*S. barnesii* SES-3	*sulba_0581* for pSB16 (fwd)	AACTTTAAGAAGGAGATATACCATGAGTAATACAGTGTTTATATTTCCTG
SB073	*S. barnesii* SES-3	*sulba_0581* for pSB16 (rev)	CCGTGTACAATACGATTACTTTCTGTTAAAACAGTGCAAGCAC
SB077	pSB15	bb-*brnE_noL2* for pSB18 (fwd)	AGATTATAAGAAGTATTTTAAACACATTAAAGTAAGCAGCTTAATTAACCTAGG
SB078	*S. barnesii* SES-3	*brnF_noK2-L5* for pSB18 (fwd)	ATATTAGTTAAGTATAAGAAGGAGATATACATATGGACCTTAAAAATGCCATAAAGC
SB079	*S. barnesii* SES-3	*brnF_noK2-L5* for pSB18 (rev)	TTACTTTAATGTGTTTAAAATACTTC
SB097	pJW75<br>pJW76	bb for pSB30, pSB31, pSB34, pSB51 (fwd)<br>bb for pSB35, pSB46 (fwd)	AAATTTGAACGCCAGCACATGG
SB098	pJW75<br>pJW76	bb for pSB30, pSB31, pSB34, pSB51 (rev)<br>bb for pSB35, pSB40, pSB46 (rev)	CATGGAATTCCTCCTGTTAGC
SB099	pSB06	*sulba_0581* for pSB30 (fwd)	TTGGGCTAACAGGAGGAATTCCATGAGTAATACAGTGTTTATATTTCCTG
SB100	pSB05	*sulba_0581* for pSB30 (rev)	AGTCCATGTGCTGGCGTTCAAATTTTTAAAACAGTGCAAGCAC
SB101	pSB23	*brnE_noL2_mcu* for pSB31 (fwd)	TTGGGCTAACAGGAGGAATTCCATGAATATCTACGCACTTTCCAGC
SB102	pSB23	*brnE_noL2_mcu* for pSB31 (rev)	AGTCCATGTGCTGGCGTTCAAATTTTTATGAGGCTGCCTGGATAATC
SB104	pSB11	*brnE_noL2_mcu* for pSB34 (fwd)	TTTTTTTGGGCTAACAGGAGGAATTCCATGAATATATACGCACTTTCATCACC
SB105	pSB11	*brnE_noL2_mcu* for pSB34 (rev)	AGACGAGTCCATGTGCTGGCGTTCAAATTTTCATGAGGCTGCCTGTATAATC
SB106	pSB10	*brnF_noK2-L5* for pSB35 (fwd)	TTTTTTTGGGCTAACAGGAGGAATTCCATGGACCTTAAAAATGCCATAAAGC
SB107	pSB10	*brnF_noK2-L5* for pSB35 (rev)	AGACGAGTCCATGTGCTGGCGTTCAAATTTTTACTTTAATGTGTTTAAAATACTTC
SB108	pRANGER-MK3<br>pSB36<br>pSB49	bb for pSB36, pSB49 (fwd)<br>bb-*brnE_noL2* for pSB37 (fwd)<br>bb-*brnE_noL2_mcu* for pSB50 (fwd)	ATGAGTCGCTAGCGACG
SB109	pRANGER-MK3	bb for pSB36, pSB49 (rev)	CATATGAGAATCTCCTTCTCTAGC

SB110	pSB34	*brnE_noL2* for pSB36 (fwd)	TTTTGGGCTAGAGAAGGAGATTCTCATATGAATATATACGCACTTTCATCACC
SB111	pSB34	*brnE_noL2* for pSB36 (rev)	GGAGTCTGAGGCTCGTCGCTAGCGACTCATTCATGAGGCTGCCTGTATAATC
SB112	pSB36	bb-*brnE_noL2* for pSB37 (rev)	TCATGAGGCTGCCTGTATAATC
SB113	pSB35	*araBAD-brnF_noK2-L5* for pSB37 (fwd)	TTTATCATGATTATACAGGCAGCCTCATGAATACTCCCGCCATTCAG
SB114	pSB35	*araBAD-brnF_noK2-L5* for pSB37 (rev)	GGAGTCTGAGGCTCGTCGCTAGCGACTCATTTACTTTAATGTGTTTAAAATACTTC
SB122	pSB26	bb for pSB40 (fwd)	TTGGGCTAACAGGAGGAATTCCATGAAATTTGAACGCCAGCACATGG
SB131	pSB32	*brnF_noK2-L5_mcu* for pSB46 (fwd)	TTGGGCTAACAGGAGGAATTCCATGGACCTGAAAAATGCCATTAAACC
SB132	pSB32	*brnF_noK2-L5_mcu* for pSB46 (rev)	AGTCCATGTGCTGGCGTTCAAATTTTTATTTGAGGGTGTTTAAAATGGAG
SB133	pSB46<br>pSB30	bb-*brnF_noK2-L5_mcu* for pSB48 (fwd)<br>bb for pSB52 (fwd)	TTGAACGCCAGCACATG
SB134	pSB46	bb-*brnF_noK2-L5_mcu* for pSB48 (rev)	TTATTTGAGGGTGTTTAAAATGGAGC
SB137	pSB31	*brnE_noL2_mcu* for pSB48 (fwd)	CTCCATTTTAAACACCCTCAAATAAATACTCCCGCCATTCAGAG
SB138	pSB31	*brnE_noL2_mcu* for pSB48 (rev)	GACGAGTCCATGTGCTGGCGTTCAATTATGAGGCTGCCTGGATAATC
SB139	pSB23	*brnE_noL2* for pSB49 (fwd)	TTTTGGGCTAGAGAAGGAGATTCTCATATGAATATCTACGCACTTTCCAGC
SB140	pSB23	*brnE_noL2* for pSB49 (rev)	GGAGTCTGAGGCTCGTCGCTAGCGACTCATTTATGAGGCTGCCTGGATAATC
SB141	pSB49	bb-*brnE_noL2_mcu* for pSB50 (rev)	TTATGAGGCTGCCTGGATAATC
SB142	pSB46	*araBAD-brnF_noK2-L5_mcu* for pSB50 (rev)	GGAGTCTGAGGCTCGTCGCTAGCGACTCATTTATTTGAGGGTGTTTAAAATGGAG
SB143	pSB46	*araBAD-brnF_noK2-L5_mcu* for pSB50 (fwd)	TTCATCATGATTATCCAGGCAGCCTCATAAATACTCCCGCCATTCAG
SB144	pSB07	*brnI* for pSB51 (fwd)	TTGGGCTAACAGGAGGAATTCCATGCACCTTGAAACTGTCTTACTAC
SB145	pSB07	*brnI* for pSB51 (rev)	AGTCCATGTGCTGGCGTTCAAATTTTTAATCTAAAGCCCGTAATTCG
SB146	pSB30	bb for pSB52 (rev)	TTAAAACAGTGCAAGCACTTCTTC
SB147	pSB51	*araBAD-brnI* for pSB52 (fwd)	AGAAGAAGTGCTTGCACTGTTTTAAATACTCCCGCCATTCAGAG
SB148	pSB51	*araBAD-brnI* for pSB52 (rev)	GACGAGTCCATGTGCTGGCGTTCAATTAATCTAAAGCCCGTAATTCGC

Table S4. Protein sequences used for validation *trans*-AT working candidate Sulba_0581. All enzymes except, BrnE_Cstart transfer a malonyl extender unit.

Protein	Description	Protein ID	Producer	Compound
KirAVI_AT2	*cis*-AT	CAN89636.1 (MIBiG)	*Streptomyces collinus* TU 365	Kirromycin
RifA_AT2	*cis*-AT	AAC01710.1 (MIBiG)	*Amycolatopsis mediterranei* S699	Rifamycin
Plu1880_AT	*cis*-AT	AXG49819.1 (MIBiG)	*Photorhabdus laumondii* TT01	Syrbactin
KirCI_AT1	*trans*-AT	CAN89639.1 (MIBiG)	*Streptomyces collinus* TU 365	Kirromycin
MlnA_AT	*trans*-AT	CAG23963.1 (MIBiG)	*Bacillus velezensis* FZB42	Macrolactin
PedD_AT	*trans*-AT	AAS47563.1 (MIBiG)	Unknown *Paederus* ssp. symbiont	Pederin
BacC_AT	*trans*-AT	CAG23950.2 (MIBiG)	*Bacillus velezensis* FZB42	Bacillaene
RhiG_AT1	*trans*-AT	CAL69887.1 (MIBiG)	*Mycetohabitans rhizoxinica* HKI 454	Rhizoxin
RhiG_AT2	*trans*-AT	CAL69887.1 (MIBiG)	*Mycetohabitans rhizoxinica* HKI 454	Rhizoxin
MisG_AT	*trans*-AT	AKQ22695.1 (MIBiG)	*Candidatus Entotheonella* sp.	Misakinolide
RizA_AT	*trans*-AT	CCA89325.1 (MIBiG)	*Stigmatella aurantiaca* Sg a15	Rhizopodin
RizF_AT	*trans*-AT	CCA89330.1 (MIBiG)	*Streptomyces collinus* TU 365	Rhizopodin
ThaC_AT1	*trans*-AT	ABC34740.1 (MIBiG)	*Bacillus velezensis* FZB42	Thailandamide
FabD_Ecoli	*trans*-AT	P0AAI9 (UniProt)	*Escherichia coli* K-12 MG1655	Fatty acid
FabD_Bsub	*trans*-AT	P71019 (UniProt)	*Bacillus subtilis* strain 168	Fatty acid
FabD_Rqing	*trans*-AT	TDL72579.1 (NCBI)	*Rhodococcus qingshengii* S-E5	Fatty acid
BrnE_Cstart	$C_{starter}$	AFL68053.1 (MIBiG)	*Sulfurospirillum barnesii* SES-3	Barnesin A

Table S5. BLASTp search of selected *trans*- and *cis*-AT query sequences against the *S. barnesii* SES-3 genome. Except Plu1880_AT and PedD_At each individual search resulted only in the listed hit. Query seq. = query sequence. Per. Ident = percentage identity.

	BLASTp results				
**Query seq.**	**Protein ID (NCBI)**	**Description**	**Query Cover**	**E value**	**Per. Ident**
KirAVI_AT2	WP_0147688770.1	ACP S-malonyltransferase	97 %	2e-48	37.81 %
RifA_AT2	WP_0147688770.1	ACP S-malonyltransferase	98 %	5e-22	27.36 %
Plu1880_AT		no hits			
KirCI_AT1	WP_0147688770.1	ACP S-malonyltransferase	100 %	3e-42	36.11 %
MlnA_AT	WP_0147688770.1	ACP S-malonyltransferase	99 %	6e-48	35.79 %
PedD_AT		no hits			
BacC_AT	WP_0147688770.1	ACP S-malonyltransferase	97 %	8e-43	34.62 %
RhiG_AT1	WP_0147688770.1	ACP S-malonyltransferase	59 %	1e-11	27.32 %
RhiG_AT2	WP_0147688770.1	ACP S-malonyltransferase	98 %	2e-32	30.88 %
MisG_AT	WP_0147688770.1	ACP S-malonyltransferase	100 %	2e-38	33.22 %
RizA_AT	WP_0147688770.1	ACP S-malonyltransferase	100 %	4e-42	33.80 %
RizF_AT	WP_0147688770.1	ACP S-malonyltransferase	94 %	1e-38	32.60 %
ThaC_AT1	WP_0147688770.1	ACP S-malonyltransferase	64 %	4e-09	27.51 %

Supplementary Figures

Sulba_0581 : 1 M S N T V F I F P … F * 924
BrnE : 1 M L N I Y A L S S … S * 2202
BrnF : 1 M K K K L D L K N … L * 1611
BrnI : 1 M H L E T V L L R … D * 212

Figure S1. N-terminal amino acid residues of the four biosynthetic enzymes required for barnesin A production. Highlighted in red are amino acids residues affected by the N-end rule. Codons encoding for the highlighted amino acid residues were removed from the respective gene sequences in expression plasmids.

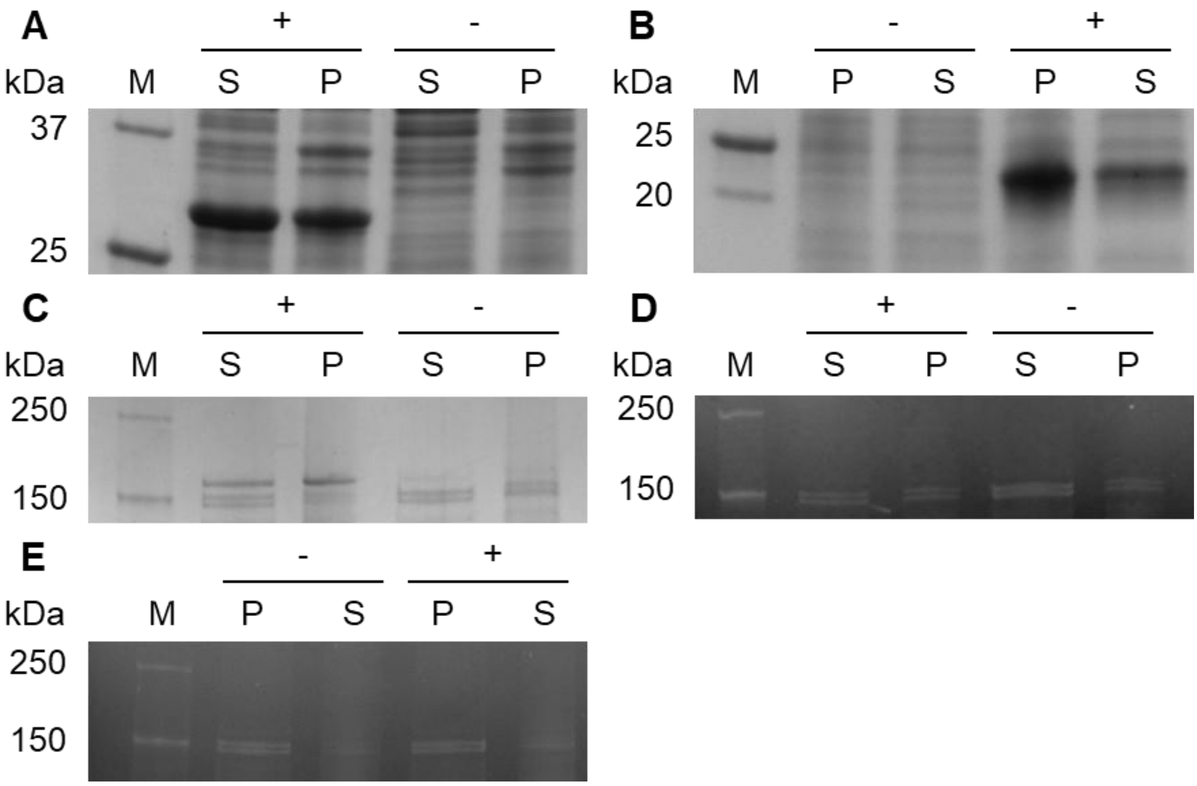

Figure S2. SDS–PAGE analyses of heterologous expression experiments using *E. coli* BL21(DE3).
A Expression of *sulba_0581* (26 °C, 1 d; 33.6 kDa), **B** expression of *brnI* (26 °C, 1 d; 24.2 kDa), **C** expression of *brnF* (18 °C, 3 d; 178.2 kDa), **D** expression of *brnE* (18 °C, 3 d; 247.8 kDa) and **E** expression of *brnE_mcu* (18 °C, 3 d; 247.8 kDa). + indicates addition of inductor IPTG at 0.1 mM. - indicates negative control without addition of inductor. M = reference marker, S = soluble fraction, P = pellet fraction.

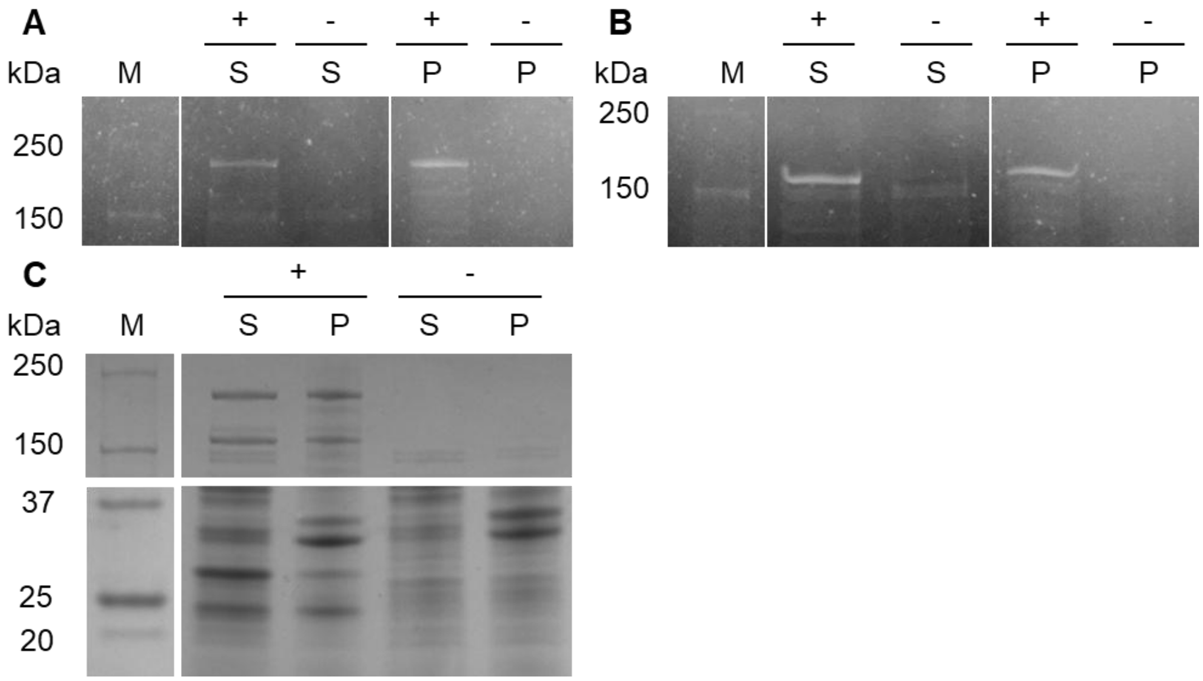

Figure S3. SDS–PAGE analyses of heterologous expression experiments in *E. coli* DH10B. A Expression of *brnE_mcu* (247.8 kDa) and **B** expression of *brnF_mcu* (178.2 kDa) at 18 °C for 3 d in *E. coli* DH10B::*mtaA*. **C** Co-expression of *brnE_mcu* (247.8 kDa), *brnF_mcu* (178.2 kDa), *sulba_0581* (33.6 kDa) and *brnI* (24.2 kDa) at 15 °C for 5 d in *E. coli* DH10B.+ indicates addition of inducer *L*-arabinose at 0.02 %. - indicates negative control without addition of inducer. M = reference marker, S = soluble fraction, P = pellet fraction.

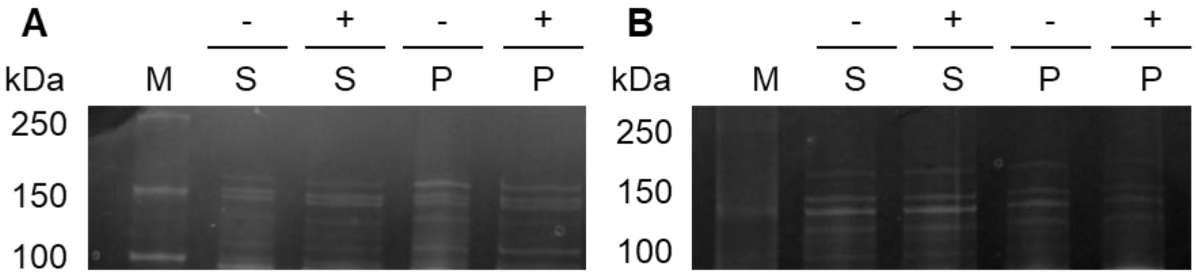

Figure S4. SDS–PAGE analyses of heterologous expression experiments in *P. protegens* Pf-5 Δ*gacA*.
A Co-expression of *brnE* and *brnF* and **B** co-expression of *brnE_mcu* and *brnF_mcu* (both 18 °C, 24 h; 247.8 kDa and 178.2 kDa). + indicates addition of inducer *L*-arabinose at 0.2 %. - indicates negative control without addition of inducer. M = reference marker, S = soluble fraction, P = pellet fraction.

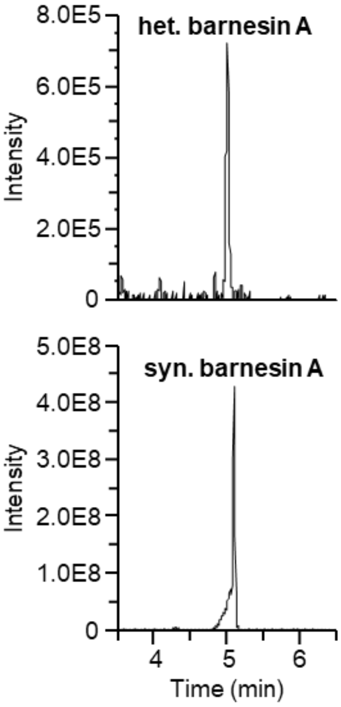

Figure S5. Retention time comparison. Retention time comparison of heterologously produced (het.) barnesin A (top) and synthetic (syn.) barnesin A (bottom; both EIC *m/z* 488.2867 $[M+H]^+$).

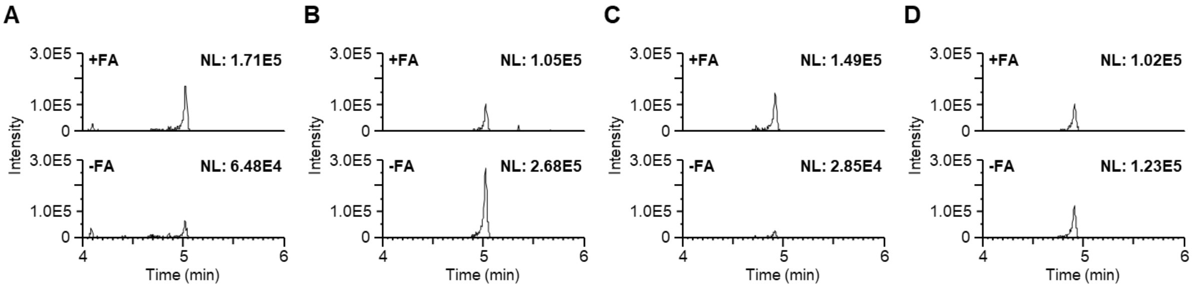

Figure S6. Influence of *trans*-2-octenoic acid supplementation on barnesin biosynthesis. EIC comparison of expression conditions with (top) and without (bottom) *trans*-2-octenoic acid supplementation. **A** barnesin A (**5**) *m/z* 488.2867 $[M+H]^+$. **B** barnesin A_1 (**6**) *m/z* 490.3024 $[M+H]^+$. **C** barnesin B (**7**) *m/z* 462.2711 $[M+H]^+$. **D** barnesin B_1 (**8**) *m/z* 464.2867 $[M+H]^+$. FA = fatty acid. NL = normalization level.

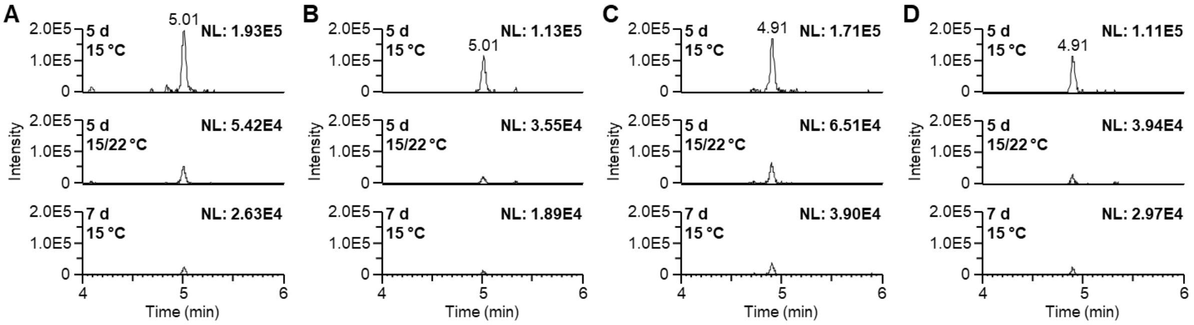

Figure S7. Efforts to increase barnesin production. EIC comparison of multiple expression conditions (top = 5 d at 15 °C; middle = 3 d at 15 °C followed by 2 d at 22 °C; bottom = 7 d at 15 °C). **A** barnesin A (**5**) *m/z* 488.2867 $[M+H]^+$. **B** barnesin A_1 (**6**) *m/z* 490.3024 $[M+H]^+$. **C** barnesin B (**7**) *m/z* 462.2711 $[M+H]^+$. **D** barnesin B_1 (**8**) *m/z* 464.2867 $[M+H]^+$. NL = normalization level.

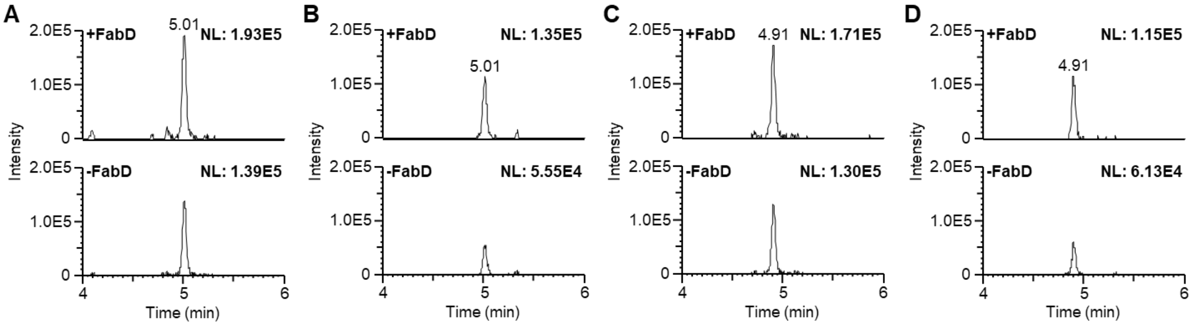

Figure S8. Influence of *sulba_0581* expression on barnesin biosynthesis. EIC comparison of expression conditions including *sulba_0581* (+FabD = *brnE_mcu*, *brnF_mcu, brnI*, *sulba_0581*; top) and conditions lacking *sulba_0581* (-FabD = *brnE_mcu*, *brnF_mcu*, *brnI*; bottom). **A** barnesin A (**5**) *m/z* 488.2867 $[M+H]^+$. **B** barnesin A_1 (**6**) *m/z* 490.3024 $[M+H]^+$. **C** barnesin B (**7**) *m/z* 462.2711 $[M+H]^+$. **D** barnesin B_1 (**8**) *m/z* 464.2867 $[M+H]^+$. NL = normalization level.

Supplementary References

[1] Tamura K, Stecher G, Kumar S. MEGA11: Molecular Evolutionary Genetics Analysis Version 11. Mol Biol Evol. 2021;38(7):3022-3027. doi: 10.1093/molbev/msab120.

[2] Lessard, J. C. Transformation of E. coli via electroporation. *Methods in enzymology* **529,** 321–327; 10.1016/B978-0-12-418687-3.00027-6 (2013).

[3] Choi KH, Kumar A, Schweizer HP. A 10-min method for preparation of highly electrocompetent Pseudomonas aeruginosa cells: application for DNA fragment transfer between chromosomes and plasmid transformation. J Microbiol Methods. 2006;64(3):391-7. doi: 10.1016/j.mimet.2005.06.001.

[4] Pfeifer BA, Admiraal SJ, Gramajo H, Cane DE, Khosla C. Biosynthesis of complex polyketides in a metabolically engineered strain of E. coli. Science. 2001;291(5509):1790-2. doi: 10.1126/science.1058092.

[5] Schimming O, Fleischhacker F, Nollmann FI, Bode HB. Yeast homologous recombination cloning leading to the novel peptides ambactin and xenolindicin. Chembiochem. 2014;15(9):1290-4. doi: 10.1002/cbic.201402065

[6] Bode E, Heinrich AK, Hirschmann M, Abebew D, Shi YN, Vo TD, Wesche F, Shi YM, Grün P, Simonyi S, Keller N, Engel Y, Wenski S, Bennet R, Beyer S, Bischoff I, Buaya A, Brandt S, Cakmak I, Çimen H, Eckstein S, Frank D, Fürst R, Gand M, Geisslinger G, Hazir S, Henke M, Heermann R, Lecaudey V, Schäfer W, Schiffmann S, Schüffler A, Schwenk R, Skaljac M, Thines E, Thines M, Ulshöfer T, Vilcinskas A, Wichelhaus TA, Bode HB. Promoter Activation in Δhfq Mutants as an Efficient Tool for Specialized Metabolite Production Enabling Direct Bioactivity Testing. Angew Chem Int Ed Engl. 2019 Dec 19;58(52):18957-18963. doi: 10.1002/anie.201910563.

[7] Klapper M, Hübner A, Ibrahim A, Wasmuth I, Borry M, Haensch VG, Zhang S, Al-Jammal WK, Suma H, Fellows Yates JA, Frangenberg J, Velsko IM, Chowdhury S, Herbst R, Bratovanov EV, Dahse HM, Horch T, Hertweck C, González Morales MR, Straus LG, Vilotijevic I, Warinner C, Stallforth P. Natural products from reconstructed bacterial genomes of the Middle and Upper Paleolithic. Science 2023, 380, 619-624.

[8] Bozhueyuek KAJ, Watzel J, Abbood N, Bode HB. Synthetic Zippers as an Enabling Tool for Engineering of Non-Ribosomal Peptide Synthetases*. Angew Chem Int Ed Engl. 2021;60(32):17531-17538. doi: 10.1002/anie.202102859.
